# Functional changes in the gut microbiota are associated with the intestinal phenotype in A20 haploinsufficiency

**DOI:** 10.1111/pai.70343

**Published:** 2026-04-16

**Authors:** Inès Elhani, Marius Bredon, Diana Enea, Aurore Desmons, Lionel Arrive, Céline Bazille, Antoine Lefevre, Achille Aouba, Adrien Bigot, Claire de Moreuil, Iria Alonso, Hélène Blasco, Laura Creusot, Camille Dupuy, Patrick Emond, Pranvera Krasniqi, Antonin Lamaziere, Cyriane Oeuvray, Dominique Rainteau, Magali Svrcek, Nathalie Rolhion, Harry Sokol, Sophie Georgin‐Lavialle

**Affiliations:** ^1^ Centre de Recherche Saint‐Antoine, CRSA, Sorbonne Université, Institut National de la Santé et de la Recherche Médicale, Gastroenterology Department, Assistance publique‐Hôpitaux de Paris, Saint‐Antoine Hospital, Paris, France Sorbonne Université Paris France; ^2^ Paris Center for Microbiome MedICIsne (PaCeMM) FHU Paris France; ^3^ Sorbonne University, Internal medicine department, Tenon hospital, CEREMAIA, AP‐HP, DMU3ID, FHU INFLAMME, INSERM UMRS1155, Paris, France & ERN RITA Sorbonne University Paris France; ^4^ Department of Pathology, Saint‐Antoine Hospital, AP‐HP Sorbonne University Paris France; ^5^ INSERM, AP‐HP, Département Metomics, Hôpital Saint Antoine, Centre de Recherche Saint‐Antoine Sorbonne Université Paris France; ^6^ Institut Curie PSL Research University, Service de radiologie, F‐92210, Saint‐Cloud, France Paris France; ^7^ Department of Pathology CHU of Caen Normandie Caen France; ^8^ R 1253, iBrain, INSERM University of Tours Tours France; ^9^ Department of Internal Medicine Caen University Hospital Caen France; ^10^ Service de Médecine Interne CHRU de Tours Tours France; ^11^ Department of Internal Medicine Brest University Hospital Brest France; ^12^ Université Paris‐Saclay, INRAE, AgroParisTech Micalis Institute Jouy‐en‐Josas France

**Keywords:** A20 haploinsufficiency, inflammatory bowel disease, microbiota

## Abstract

**Background:**

A20 haploinsufficiency (HA20) is an autoinflammatory disease driven by pathogenic variants in *TNFAIP3*, which plays a crucial role in regulating immune responses. The clinical manifestations of HA20 resemble those of inflammatory bowel disease (IBD), with prominent gastrointestinal (GI) involvement. Given the well‐established association between gut microbiota alterations and IBD, this study aimed to describe the GI involvement of HA20 patients and to investigate their fecal microbiota using shotgun sequencing and metabolomics.

**Methods:**

This study included 16 HA20 patients and 22 healthy age and sex‐matched controls. GI clinical phenotype, liver imaging, and liver and GI tissue histology were assessed. Shotgun metagenomic sequencing was performed on fecal DNA. Fecal metabolomic profiling of bile acids, short‐chain fatty acids (SCFAs), and tryptophan metabolites was performed.

**Results:**

Liver imaging revealed chronic liver disease in 3/5 patients, showing as liver dysmorphia and portal hypertension. Histological analysis showed lymphoplasmocytic infiltrate of the GI tract and the liver. The fecal microbiota of HA20 patients was characterized by marked alterations, including a reduction in microbial diversity and an increase in the pro‐inflammatory bacterium *Ruminococcus gnavus*. Microbial bile acid deconjugation and desulfation were impaired. Additionally, tryptophan metabolism was altered, with a shift towards the kynurenine pathway.

**Conclusion:**

Our results show that HA20 is associated with gut microbiota alterations and significant disruptions in metabolic pathways, particularly involving bile acids. These alterations could contribute to the chronic inflammation observed in HA20. These findings highlight the role of the gut‐liver axis and of mucosal barrier dysfunction in HA20.

**Key message**

A20 haploinsufficiency (HA20) is a genetic disease associated with hyperactivation of the NF‐κB pathway. It causes systemic inflammation and is considered to be a cause of monogenic inflammatory bowel disease. HA20 is associated with inflammatory gut dysbiosis and alterations in the bile acids metabolism that could directly contribute to the systemic and digestive inflammatory phenotype. Microbiota modulation and bile‐acids targeted therapies could improve the gut symptoms as well as systemic inflammation in HA20 patients.

## 1 INTRODUCTION

A20 haploinsufficiency is an inherited disease associated with loss‐of‐function variants of *TNFAIP3*, which encodes A20.^1^ It is a newly discovered disease with less than 190 patients described to date.^1^ HA20 is responsible for excessive innate immunity activation through the NF‐κB pathway and systemic inflammation. Patients present with recurrent and/or chronic fever and various systemic involvements. Of these, intestinal involvement is the most common, affecting 46% of described patients.^1^ Recurrent intestinal ulcerations are common and may affect the entire gastrointestinal (GI) tract. Some patients display episodes of colitis with transmural inflammation resembling Crohn's disease. In addition, 2/3 of all patients suffer from recurrent mouth ulcers.^1^ In humans, genome‐wide association studies have shown an association between *TNFAIP3* polymorphisms and inflammatory bowel disease (IBD).^2^ Moreover, altered expression of A20 correlates with disease severity in IBD.^3^ Altogether, HA20 is considered by some authors to be a monogenic IBD.^4^

Although multifactorial, the pathogenesis of IBD is closely linked to gut dysbiosis, which is also associated with systemic inflammation.^5^ These effects are mediated by direct interactions between the microbiota and the host, and by the conversion of dietary or host‐derived metabolites.^6^ Among these, bile acids, short‐chain fatty acids (SCFAs), and tryptophan metabolites can exert either pro or anti‐inflammatory effects depending on the microbial environment.^6^ For instance, SCFAs, such as butyrate, a product of fiber fermentation by gut bacteria, are known for their anti‐inflammatory properties, promoting regulatory T‐cell function and maintaining epithelial barrier integrity. Conversely, dysregulation in bile acid metabolism, often observed in IBD patients, can lead to an accumulation of pro‐inflammatory bile acid species that exacerbate intestinal inflammation.

Preliminary results from 16S sequencing have suggested dysbiosis in 16 patients with HA20 from Japan compared to their healthy family members.^7^ Interestingly, the clinical presentation of HA20 may differ according to geographic location.^1^, ^8^ Moreover, ethnicity and geographic origin influence gut microbiome composition.^9^ Therefore, in this study, we aimed to investigate in depth the GI phenotype of patients with HA20 as well as their fecal microbiota composition and functions using shotgun metagenomics sequencing and targeted metabolomics including bile acid, SCFAs, and tryptophan metabolite profiles.

## 2 METHODS

### 2.1 Patients' inclusion

A cross‐sectional study was conducted in the French reference centers for auto‐inflammatory diseases (Tenon hospital, Paris, and Caen university hospitals) from 2020 to 2023. Consenting patients with HA20 were included. Disease characteristics (mutational status, sex, age, organ involvement, and current treatment) were extracted from medical records. Patients completed a form covering stool consistency (Bristol Stool Scale),^10^ current antibiotic use, number of bowel movements per day, and presence of inflammatory symptoms (fever, joint pain, abdominal pain, muscle pain).

Participants collected stool samples at home, stored them at −20°C, and returned them to the lab within 7 days for storage at −80°C until DNA extraction. The study was approved by institutional review boards (Comité de protection des personnes Sud‐Est III, 2022‐A02073‐40; comité d'éthique de la recherche, Sorbonne Université). Twenty‐two age‐ and sex‐matched controls were selected from “Suivitheque” Biobank (Comité de Protection des Personnes Ile‐de‐France IV, IRB 00003835, Suivitheque study; registration number 2012/05NICB).

### 2.2 MRI analysis

Liver MRIs from 5 patients were retrospectively evaluated for hepatic dysmorphia, parenchyma heterogeneity, liver edge abnormalities, ascites, spleen size, portocaval shunts, portal hypertension, perfusion disorders, and vein anomalies. Liver enlargement was defined as a caudate‐to‐right lobe ratio ≥0.9. Spleen enlargement was defined as spleen length >12 cm and/or volume >480 cm^3^.

Detailed histological and microbiota analyses are described in Appendix S1.

## 3 RESULTS

### 3.1 Key patients' characteristics

Sixteen patients from 6 families of European ancestry with HA20 were studied and are described in Tables 1 and 2. Three families had been previously described.^11^, ^12^, ^13^ Median age was 29.5 [12–57]. Mouth ulcers were described in 16 patients (100%) and genital ulcers in 13 (81%). Abdominal symptoms, including pain and colitis, were noted in 13 patients (81%). Elevated liver enzymes were noted in 4 patients (25%). The median body mass index was 21.2 kg/m^2^ (range: 15.1–29.7). Comparisons between HA20 patients and healthy controls showed no significant differences in sex, age, or BMI. Interferon‐stimulated gene expression was elevated in all 5 patients in whom it was measured, with a median of 18.4 [4.4–23.2] (positive when >2.3). At the time of stool collection, five patients were taking colchicine, and six were receiving immunosuppressants. No patients received antibiotics in the month before sampling.

**TABLE 1:** Main features of patients with A20 haploinsufficiency and healthy controls.

	HA20 patients *n* = 16	Controls *n* = 22	*p*
Females	12 (71)	25 (78)	.7
Age (years)	29.5 [12–57]	38 [23–69]	.1
BMI (kg/m^2^)	21.2 [15.1–29.7]	21.4 [17–33.2]	.7
HA20 symptoms			
Mouth ulcers	16 (100)		
Genital ulcers	13 (81)		
Colitis	4 (25)		
Gastrointestinal ulcer	7 (44)		
Elevated liver enzyme	4 (25)		
Positive interferon signature	5/5 (100%)		
Median	18.4 [4.4; 23.2]		
Treatments on collection day			
Colchicine	5 (31)		
TNF‐α blockers	5 (31)		
On collection day			
Bristol stool scale	3 [3–5]		
Abdominal pain	4 (25)		
Number of stools/day	1 [1–3]		

*Note*: Values are median [range] for quantitative variables and number (%) for qualitative variables.

Abbreviations: BMI, Body max index; GI, gastrointestinal; HA20, A20 haploinsufficiency.

**TABLE 2:** Detailed clinical history of all 16 patients included.

Patient number	Family	History of mouth ulcers	History of Genital ulcers	History of gut symptoms	History of colitis/gut ulcers	Liver disease	GI symptoms on collection day	Colchicine on collection day	Other DMARDs on collection day
1	1	1	0	1	1	1	1	0	0
2	1	1	1	1	1	0	0	0	Adalimumab
3	2	1	1	1	1	0	0	1	Adalimumab
4	2	1	1	1	0	0	0	1	0
5	3	1	1	1	1	0	0	0	0
6	3	1	1	1	0	0	0	1	Azathioprine
7	3	1	1	1	0	0	0	0	0
8	4	1	1	1	1	0	0	1	0
9	4	1	1	1	1	0	1	1	Adalimumab
10	5	1	0	0	0	1	0	0	0
11	6	1	1	1	0	1	1	0	0
12	6	1	1	0	1	0	0	0	0
13	6	1	1	1	0	1	0	0	Adalimumab
14	6	1	1	1	0	0	0	0	0
15	6	1	0	0	1	0	0	0	0
16	6	1	1	1	0	1	0	0	Adalimumab

Abbreviations: DMARDs, disease‐modifying anti‐rheumatism drugs; GI, gastrointestinal.

### 3.2 HA20 patients show an MRI‐pattern of chronic liver disease

**FIGURE 1:**
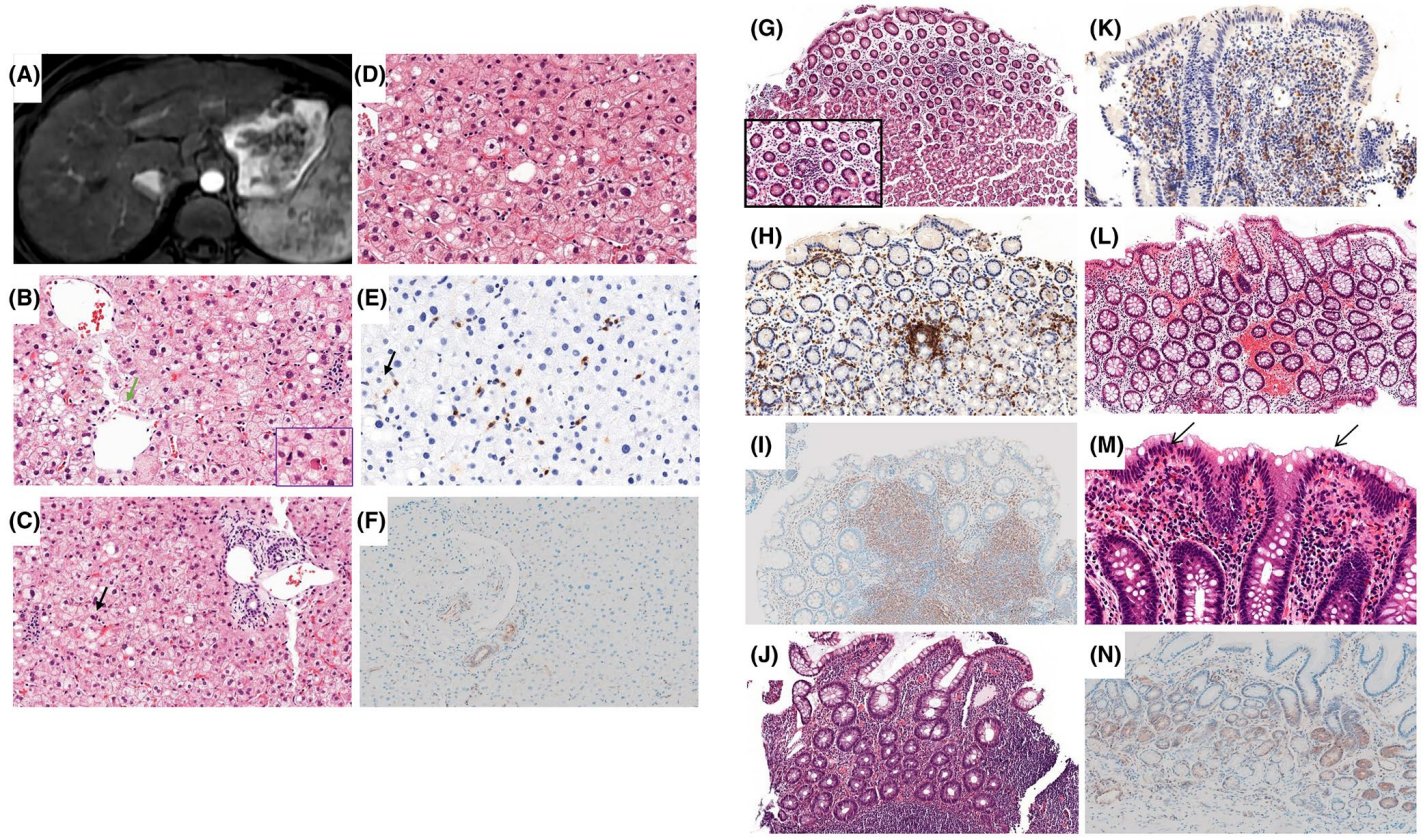
Morphological analysis of the liver in patients with A20 haploinsufficiency. (A) Liver MRI displaying characteristics of chronic liver disease. The liver exhibits irregular margins, dysmorphic shape, and a multinodular pattern. Splenomegaly is also present, suggesting the presence of portal hypertension. (B, C) The liver architecture is normal, with no fibrosis. There is minimal portal inflammation without interface hepatitis in a few portal structures (HE stain, intermediate power view). There are a few hepatocyte ballooning lesions (green arrow), rare inflammatory lobular foci (black arrows), and few apoptotic bodies (detail). (D, E) Sinusoid lymphocytic inflammatory infiltrate positive for CD8 antibodies. (F) Immunohistochemistry shows a slight labeling with NF‐κB p65 in rare lymphocytes circulating within the sinusoids. (G) Fundic biopsies revealing lesions of gastritis with a predominant lymphocytic inflammatory infiltrate (low and intermediate power view). Immunohistochemistry shows CD8‐positive cells (H) and intermediate NF‐κB p65 cytoplasmic staining (I) in the inflammatory infiltrate. (J) Histological examination of the ileal mucosa reveals architectural distortion and atrophic villi, marked lamina propria inflammation, and ulceration (HE stain, intermediate magnification). (K) Immunohistochemistry shows CD8‐positive cells in the inflammatory infiltrate. (L) Histological examination at low power view showing colonic mucosa with normal glandular architecture, lamina propria edema and congestion, and frequent lymphoid nodules. (M, N) Focally, eosinophils penetrated the surface epithelium (arrows; intermediate power view, HE stain).

Liver MRI findings were assessed in five patients from three families. The MRI results were normal for two patients, with no evidence of structural or functional abnormalities observed. However, the remaining three patients exhibited significant liver dysmorphia, characterized by irregular liver contour and altered lobar anatomy. Among these three patients, two presented with a pattern of chronic liver disease that included heterogeneous liver parenchyma, porto‐systemic collateral vessels (portocaval derivation), altered hepatic blood flow and signs of portal hypertension (Figure 1A). They also presented with an enlarged spleen with a spleen length of 163 and 160 cm and a spleen volume of 879 and 1058 cm^3^, respectively. The liver was enlarged in two patients, with a caudate‐to‐right lobe ratio of 1.5 and 1.2, respectively. None of the patients had abnormalities of the hepatic veins, portal veins or bile ducts.

### 3.3 HA20 patients display histological features of liver gastro‐intestinal tract inflammation

Liver tissue was available from 1 patient, revealing a pattern of steatohepatitis with low‐grade portal inflammation, mild lobular inflammation, and low‐grade steatosis (SAF score S1A2F0). There was no bile duct inflammatory infiltrate (Figure 1B–E). Immunohistochemical analysis showed a higher number of CD‐8 positive cells (Figure S1A,B) and a slight labeling with NF‐κB p65 in rare lymphocytes circulating within the sinusoids (Figure 1F).

Stomach tissue was available from 3 patients, all of whom showed a lymphoplasmacytic infiltrate (Figure 1G,H; Figure S1C,D). CD8‐positive T cells predominated in the infiltrate (Figure S1E–H). Additionally, one patient exhibited a neutrophilic infiltrate. Weak to intermediate cytoplasmic labeling of the chorion was observed in two samples using the anti‐NF‐κB p65 antibody, while no staining was detected in one sample (Figure 1I).

Ileal tissue was available from 1 patient, who displayed severe neutrophilic and lymphocytic infiltrates of predominantly CD4‐positive T cells (Figure 1J,K) with rare CD8‐positive T cells (Figure S1I). Mild erosions were observed, but no evidence of apoptosis. MUC2 immunostaining was normal and no Paneth cell apoptosis was observed. NF‐κB immunostaining revealed very weak and focal staining of the inflammatory infiltrate and lymphoid follicles.

Colon tissue was available from 4 patients. B‐cell lymphoid aggregates were observed in 3 patients, one of whom also showed an eosinophilic infiltrate (Figure 1L,M). One patient additionally displayed a lymphoplasmocytic infiltrate of predominantly CD4‐positive T cells (Figure 1K). Rare apoptosis was noted in 1 patient (Figure S1K). MUC2 immunostaining was normal in all cases (Figure S1E). NF‐κB p‐65 immunostaining revealed intermediate cytoplasmic staining of the inflammatory infiltrate within the chorion in four samples, with associated staining of lymphoid follicles observed in two of these samples (Figure 1N).

### 3.4 HA20 patients display significantly different microbiota composition compared to healthy subjects

**FIGURE 2:**
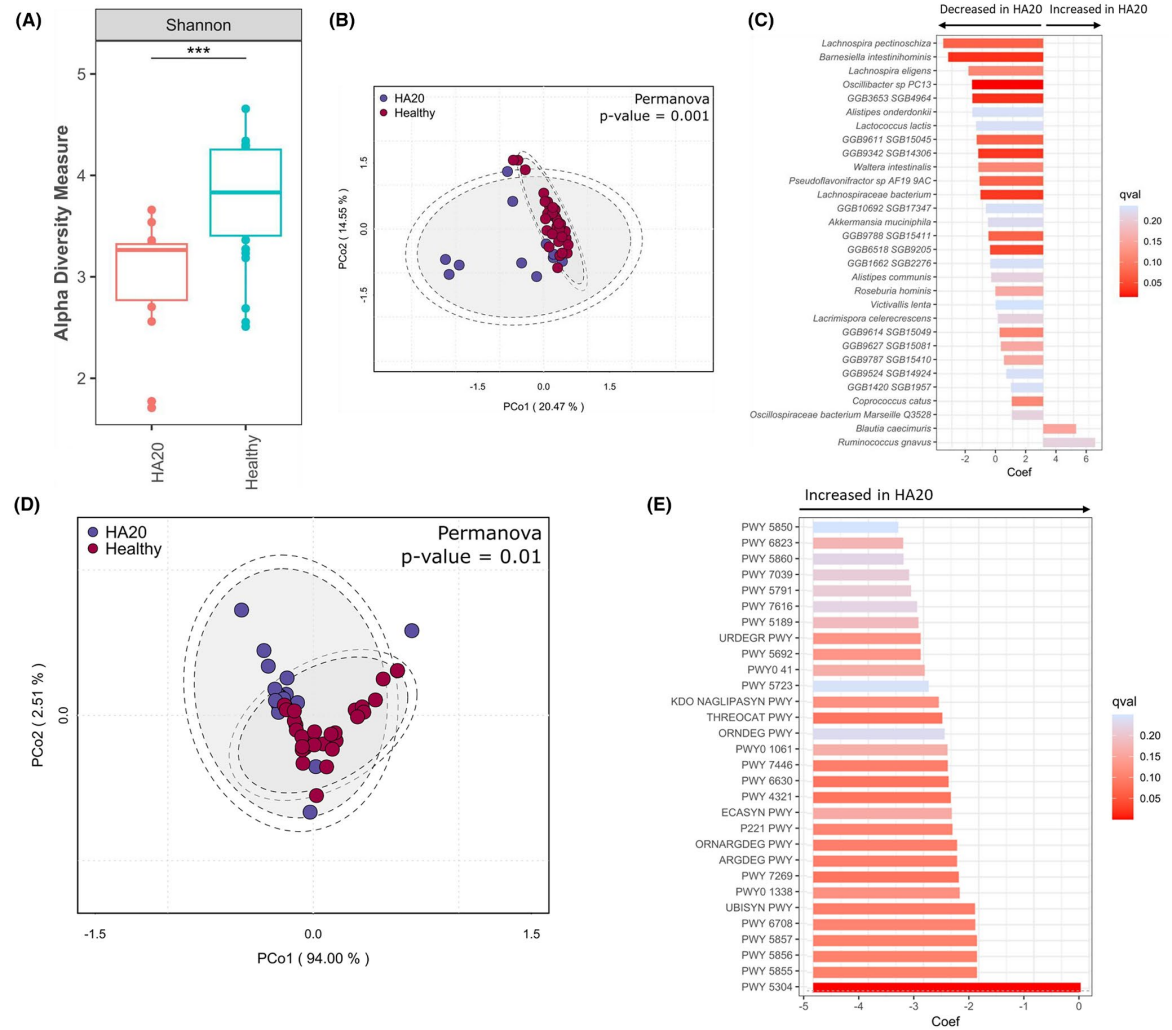
Gut microbiota taxonomic composition and functional profiles in patients with A20 haploinsufficiency. (A) Boxplot comparing the alpha diversity (Shannon Index) between A20 patients (HA20) and healthy controls. (B) Principal Coordinates Analysis (PCoA) based on Bray–Curtis dissimilarity. (C) Bar plot showing the differential abundance of key microbial taxa between HA20 patients and healthy subjects, identified using MaAsLin2. (D) Principal Coordinates Analysis (PCoA) of functional pathway profiles. (E) Bar plot showing the differential abundance of functional pathways between HA20 patients and healthy subjects, identified using MaAsLin2.

Microbial diversity, as measured by the Shannon index, was found to be significantly decreased in patients with HA20 (*p* < .001, Figure 2A). Separate clusters were identified in the beta diversity PCoA plot, indicating a different distribution of gut microbiota between HA20 patients and healthy subjects (*p =* .001; Figure 2B).

Estimated taxonomy of the bacterial species from species‐level genome bins is detailed in Table S1. Multivariate Association with Linear Models Multivariate 2 (MaAsLin2) pointed out a decrease in the relative abundance of 28 bacteria and an increase of sequences from 2 bacteria in HA20 patients (Figure 2C). Eleven decreased bacteria had known anti‐inflammatory properties, including butyrate production (*Roseburia_hominis*,^14^ *Oscillospiraceae_sp*,^15^ *Coprococcus_catus*,^16^ *Eubacterium_ramulus*,^17^ *pseudoflavonifractor* spp,^18^ *Roseburia_hominis*,^19^ and *Akkermansia_muciniphila* ^20^) and gut barrier enhancement (*Akkermansia_muciniphila*,^21^ *Roseburia hominis* ^14^). Conversely, sequences associated with *Ruminococcus gnavus*, which is a known pro‐inflammatory mucin‐degrading bacterium, were increased in HA20 patients.^22^

Altogether, these data suggest that the gut microbiota of HA20 patients was distinct from that of healthy controls and was associated with a depletion in anti‐inflammatory taxa and an enrichment of pro‐inflammatory bacteria.

### 3.5 Functional analyses of shotgun metagenomics suggest higher activity of metabolic pathways involved in inflammation and gut barrier disruption in HA20

Functional analyses revealed a significantly different profile of genetic sequences related to metabolic pathways in HA20 patients (*p =* .01, Figure 2D). Correspondence between identification number and metabolic pathway is reported in Table S2.

In HA20, several pathways involved in gut barrier alterations were increased. It is the case of the superpathway of sulfur oxidation (PWY‐53014), which may lead to the production of the toxic hydrogen sulfide, and the superpathway of L‐threonine degradation (THREOCAT PWY), which is a major component of mucin. Two microbial L‐arginine degradation pathways were increased (ARGDEG PWY and ORNARGDEG PWY). These two pathways lead to the production of putrescine that can be toxic when present in large quantities. Moreover, the pathways for biosynthesis of the pro‐inflammatory bacterial components enterobacterial common antigen (ECASYN PWY) and lipopolysaccharide‐associated lipid‐A (KDO‐NAGLIPASYN‐PWY) were increased. HA20 patients also displayed increased relative abundance of metabolic pathways involved in molybdopterin synthesis (PWY‐6823) and quinol biosynthesis (PWY‐5850, PWY‐5860, PWY‐6708, PWY‐5857, PWY‐5856, PWY‐5855, and UBISYN PWY) (Figure 2E). The molybdopterin pathway catalyzes the reaction transforming nitrate to nitrite and nitric oxide. Nitric oxide overproduction in the Gi tract is associated with altered gut barrier.^23^ Quinol biosynthesis is a major source of energy through the mitochondrial energy chain and increased abundance of quinol biosynthesis related pathways has been described in IBD.^24^, ^25^

### 3.6 Bile acids profile suggests microbiota‐associated defects in bile acid in HA20 patients

**FIGURE 3:**
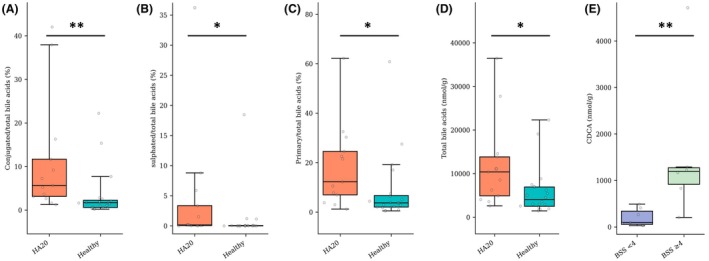
Fecal bile acid profile in patients with A20 haploinsufficiency (HA20) compared to healthy subjects. (A) Conjugated to total fecal bile acids ratio (%). (B) Sulphated to total fecal bile acids ratio (%). (C) Primary to total fecal bile acids ratio (%). (D) Total fecal bile acids (nmol/g of dry stool). (E) Fecal Chenodeoxycholic acid (CDCA) levels in HA20 patients according to the Bristol stool scale (BSS).

**FIGURE 4:**
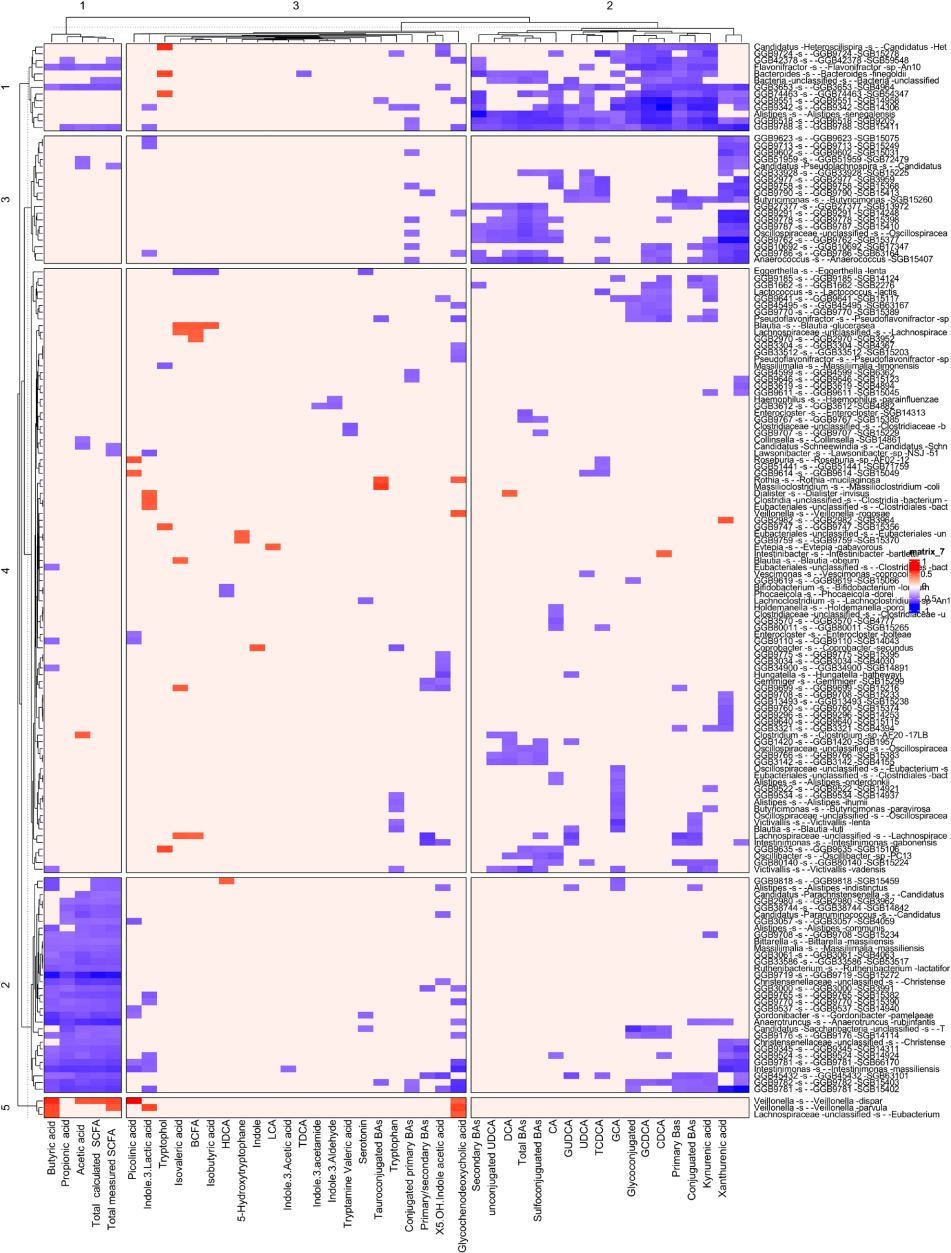
Correlation between fecal metabolites and gut microbiota in patients with A20 haploinsufficiency. SCFA, Short‐chain fatty acid; BCFA, Branched‐chain fatty acid; BAs, Bile acids; HDCA, Hyodeoxycholic acid; LCA, Lithocholic acid; TDCA, Taurodeoxycholic acid; GDCA, Glycodeoxycholic acid; DCA, Deoxycholic acid; CA, Cholic acid; GUDCA, Glycoursodeoxycholic acid; UDCA, Ursodeoxycholic Acid; TCDCA, Taurochenodeoxycholic acid; GCA, Glycocholic acid; GCDCA, Glycochenodeoxycholic acid; CDCA, Chenodeoxycholic acid.

Fecal bile acids were profiled in 13 patients and 20 healthy subjects. The proportion of conjugated (5.7% vs. 1.7%, *p =* .002) and sulphated (0.16% vs. 0.01%, *p =* .003) bile acids (BA) were significantly increased in HA20 patients, suggesting a defect in BA deconjugation and desulfation (Figures 3A and 4B). In addition, the proportion of primary BA was significantly increased in HA20 patients, reflecting a lack of transformation into secondary BA by the intestinal microbiota (12.3% vs. 3.7%, *p =* .01) (Figure 3C). Finally, global fecal BA concentration was significantly higher in HA20 patients, suggesting a deficit in the ileal absorption of BA (10389.3 vs. 4035.91 nmol/g, *p =* .01) (Figure 3D). Moreover, the level of Chenodeoxycholic acid (CDCA), one major primary BA, was significantly higher in patients with looser stool (BSS ≥ 4), suggesting that CDCA is directly associated with stool consistency (1196.3 VS 96.2 nmol/mol, *p =* .008, Figure 3E).

Negative correlations were observed between the abundance of Firmicutes and Bacteroidita phyla and the levels of conjugated bile acids. Moreover, 26 bacterial species, including 20 that belonged to the phylum Firmicutes, were negatively associated with levels of primary bile acids (Figure 4). However, among fully annotated bacteria, none were known to possess 7‐α‐dehydroxylation activity, which carries transformation of primary bile acids to secondary bile acids.

### 3.7 HA20 patients harbor altered fecal short‐chain fatty acids (SCFA) profile

**FIGURE 5:**
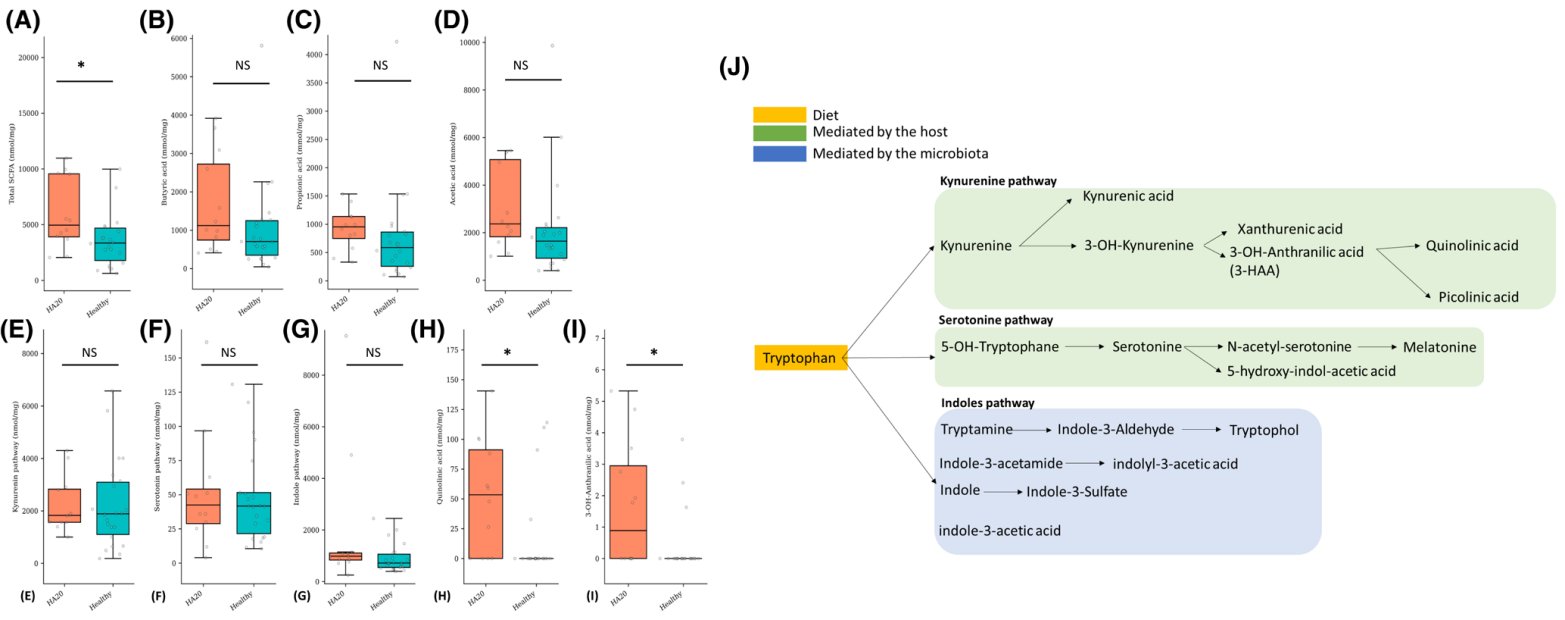
Fecal short‐chain fatty acid and tryptophan metabolite profiles in patients with A20 haploinsufficiency (HA20) compared to healthy controls. (A) Total fecal SCFA (nmol/mg) (B) Butyrate to total fecal SCFA ratio (%) (C) Propionate to total fecal SCFA (%) (D) Acetate to fecal total SCFA (%) (E) Total fecal metabolites of the (E) kynurenin pathway (F) serotonin pathway and (G) Indole pathway (H) Fecal quinolinic acid (nmol/mg) (I) Fecal 3‐OH‐Anthranilic acid (nmol/mg). (J) Overview of the metabolic pathway derived from dietary tryptophan.

Global Fecal SCFA concentrations were significantly higher in HA20 patients compared with healthy subjects (*p =* .04). The levels of individual SCFAs were not significantly different, suggesting that no single SCFA was more affected (Figure 5A–E). The interpretation of fecal SCFA level is complex as these molecules are produced by the microbiome but are also massively absorbed. Thus, the fecal levels do not represent the luminal production, especially in proximal colon or terminal ileum. The increased levels of fecal SCFA in HA20 patients, together with the altered microbiota diversity and composition and the intestinal inflammation, suggest a SCFA absorption defect rather than an increased production.

Fecal butyrate levels showed a positive correlation with *Eubacterium rectale*, a well‐known butyrate producer. Interestingly, butyrate levels also correlated with two members of the *Veillonella* genus, which are not typically associated with butyrate production (Figure 3). Rather, these bacteria, including *Veillonella dispar*, are recognized as acetate producers. In HA20 patients, *V. dispar* specifically exhibited a positive correlation with fecal acetate levels.

### 3.8 Tryptophan profile suggests diversion of dietary tryptophan to the kynurenine pathway in HA20 patients

When metabolites from the kynurenine pathway were summed, no significant differences were observed between HA20 patients and healthy subjects (Figure 5F). Same results were obtained with serotonin, and indole pathways (Figure 5G,H). However, within the kynurenine pathway, levels of quinolinic acid (Figure 5I) and 3‐HAA (Figure 5J) were significantly higher in HA20 patients.

Surprisingly, *Coprobacter secundus* was positively correlated to fecal levels of indoles, although it is not a known indole producer.

## 4 DISCUSSION

In this study, we described the digestive involvements of HA20 patients and analyzed the microbiota composition of 16 patients with HA20 compared to 22 healthy subjects. We found that patients displayed a non‐specific pattern of gastrointestinal inflammation and chronic liver disease. Moreover, we found microbiota alterations in patients with HA20, including the decrease of anti‐inflammatory bacteria and the expansion of the pro‐inflammatory bacteria such as *Ruminococcus gnavus*. These changes are associated with altered bile acid, SCFA, and tryptophan metabolism.

As previously described, HA20 is associated with a nonspecific histological pattern of intestinal inflammation. Notably, the analyses did not show a typical pattern of Crohn's disease or ulcerative colitis, with the absence of crypt alterations and granuloma. Given the data from murine models of TNFAIP3 insufficiency, we would have expected histological clues of a weak gastrointestinal barrier with increased apoptosis and decreased Muc‐2 immunostaining.^26^ However, these murine models do not fully replicate HA20 pathogenesis, as colonic cells completely lack A20 expression, whereas patients typically express about 50% of the normal protein due to one functional gene. Consequently, patients may produce enough A20 to maintain homeostasis under normal conditions but may be insufficient after an external insult.

IBD is associated with hepatobiliary abnormalities in 4.7%–29% of patients. Specifically, 2%–14% of IBD patients present with primary sclerosing cholangitis (PSC), which is associated with the inflammatory infiltrate and fibrosis of intra‐hepatic bile ducts.^27^ IBD and PSC are believed to develop synergistically through the gut‐liver axis. In this model, dysbiosis observed in IBD patients increases the translocation of inflammatory components into the hepatic circulation, contributing to inflammatory infiltration of the liver parenchyma. Conversely, the resulting liver inflammation disrupts bile acid metabolism, further exacerbating gut dysbiosis and creating a vicious cycle that drives the progression of both conditions.^28^ Because liver disease was described in up to 10% of HA20 patients, we had hypothesized that patients may exhibit a pattern resembling PSC due to dysbiosis and increased translocation. However, no patient displayed auto‐antibodies specific to PSC. Moreover, there was no histological nor MRI bile duct alterations in HA20 patients, which suggests that liver disease in HA20 differs from that of IBD. These results are concordant with the histological analysis of the liver and digestive tract previously described in HA20 patients.^11^ Interestingly, A20 is a well‐known hepatoprotective agent due to its anti‐inflammatory and anti‐apoptotic properties. Indeed, murine models of liver A20 deficiency are characterized by spontaneous chronic liver inflammation.^29^ Therefore, A20 haploinsufficiency in the liver may cause the non‐specific liver inflammation that patients displayed.

We observed a marked reduction in microbial diversity among HA20 patients compared to healthy controls. The composition shift in HA20 patients was marked by an overrepresentation of *R. gnavus*. This bacterium is characterized by its mucin‐degradating properties, which is the main component of the gut barrier. Therefore, it is thought to cause gut barrier disruption.^30^ In addition, the fecal microbiota of HA20 patients was depleted of anti‐inflammatory taxa, including some associated with gut barrier function. Gut barrier alteration is a major event in the development of IBD.^31^ It causes the abnormal contact of bacteria with the gut mucosa, which may instigate inflammatory reactions. Moreover, the passage of pro‐inflammatory bacterial components and/or secondary metabolites into the bloodstream may cause or worsen systemic inflammation.^31^

These results are consistent with Toyofuku et al., who had also reported dysbiosis in patients with HA20.^7^ However, the bacterial profile reported differed from our results, particularly with a decrease of *Ruminococcus*, which could be explained by the different population studied but also by the use of 16S sequencing, unlike the shotgun approach used in our study, allowing a better analysis resolution. In addition, Ruminococcus species were not detailed, and there could be an increase in the abundance of *R. gnavus* concomitant with a global decrease in the abundance of other Ruminococcus species. A20 deficiency in intestinal epithelial cells has already been linked to colitis in murine models.^26^ Dysfunction of the gut barrier, due to increased apoptosis, decreased mucus production and microbial antigen degradation, occurs early.^26^ Along with it are described microbiota changes, associating bacterial invasion of the mucosa and decreased bacterial diversity.^26^ Interestingly, microbiota transfer from A20‐deficient mice to wild‐type mice leads to higher sensitivity to DSS‐induced colitis, highlighting the synergic role of A20 deficiency and dysbiosis in the development of colitis.^32^

Bile acid metabolism alterations were associated with gastrointestinal symptoms in HA20 patients in this study. Every microbiota‐dependent step was altered. Particularly, deconjugation and transformation into secondary bile acids were associated with the depletion of Firmicutes. CDCA is a primary bile acid that possesses detergent properties that stimulate fluid secretion, mucosal permeability, and gut motility. Therefore, its abnormal persistence may cause BA diarrhea.^33^ Moreover, BA malabsorption may underactivate FXR receptor, which is a modulator of NF‐kb.^34^ BA‐based treatments, such as BA sequestrants, are under investigation in the treatment of BA diarrhea and may be a lead for the treatment of diarrhea in HA20 patients in the future.^33^

Unexpectedly, fecal SCFA was increased in HA20 patients. In IBD, fecal concentration of SCFA has been consistently shown to be decreased.^35^ Especially, butyrate is an anti‐inflammatory metabolite that acts as the main source of energy for colonocytes and promotes gut barrier function.^36^ In our study, while some butyrate‐producing bacteria were depleted in HA20 patients, fecal butyrate concentration was increased, which suggests absorption deficiency. Indeed, inflammatory cytokines, such as TNF‐α, may downregulate SCFA receptor expression.^37^ Therefore, the inflammatory state of HA20 patients may cause functional butyrate deficiency and promote gut barrier alterations.

Perspectives of microbiota‐targeted treatment in HA20 are appealing. The inflammatory properties of microbiota from A20‐deficient mice suggest that its modulation could improve GI symptoms. However, results from clinical studies involving patients with rheumatic diseases are equivocal and seem to show biological improvement at best.^38^ While most studies have been conducted using readily available probiotics (Lactobacillus, Bacillus, and Bifidobacterium genera), the use of specific bacteria tailored to the dysbiosis profile should be evaluated. Finally, it is unlikely that supplementation of one bacteria alone could alleviate symptoms as the whole host‐microbiota network is altered in HA20 patients.

HA20 is associated with an increased production of inflammatory cytokines, including interleukin‐1β and TNF‐α. In addition, several studies have reported increased interferon signature in HA20 patients.^1^ Here, HA20 patients showed a diversion of dietary tryptophan to the kynurenic pathway, which was associated with increased fecal levels of the neurotoxic metabolite picolinic acid. Interestingly, interferon‐γ induces the expression of indolamine 2,3‐dioxygenase (IDO1), which mediates the entry of tryptophan into the kynurenine pathway. Therefore, the inflammatory phenotype of HA20 could be directly responsible for the accumulation of neurotoxic metabolites, particularly through high levels of interferon‐γ. Modulation of interferon‐γ could be an interesting approach for the treatment of HA20. Current treatments for gastrointestinal involvement in HA20 are not codified and are adapted from treatments commonly used in IBD. Oral 5‐aminosalicylic acid and TNF‐α blockers are most commonly used, with heterogeneous responses.^1^ In IBD, JAK inhibitors have been recently proposed to treat patients resistant to TNF‐α blockers.^39^ Moreover, they have been used successfully to treat neurological involvements, uveitis and arthritis in HA20.^1^ In this study, one patient treated with TNF‐α displayed sustained gastrointestinal inflammation, which was successfully alleviated. Therefore, JAK inhibitors may be an alternative treatment for HA20 with gastrointestinal involvements.

In conclusion, this study identifies distinct gastrointestinal and liver involvement in HA20 patients, marked by reduced microbial diversity and an increase in pro‐inflammatory bacteria *like Ruminococcus gnavus*. These changes suggest a compromised gut barrier and altered bile acid metabolism contributing to inflammation. Despite some similarities to IBD, the liver pathology in HA20 appears unique, highlighting the potential for microbiota‐targeted therapies and the need for tailored treatment strategies in these patients.

## AUTHOR CONTRIBUTIONS

**Inès Elhani:** Formal analysis; writing – original draft; investigation; funding acquisition; data curation. **Marius Bredon:** Formal analysis; software; data curation; writing – review and editing; methodology; investigation. **Diana Enea:** Formal analysis; writing – review and editing. **Aurore Desmons:** Formal analysis; writing – review and editing. **Lionel Arrive:** Formal analysis; writing – review and editing. **Céline Bazille:** Formal analysis; writing – review and editing. **Antoine Lefevre:** Formal analysis; writing – review and editing. **Achille Aouba:** Formal analysis; writing – review and editing; investigation. **Adrien Bigot:** Writing – review and editing; investigation. **Claire de Moreuil:** Investigation; writing – review and editing. **Iria Alonso:** Formal analysis; writing – review and editing. **Hélène Blasco:** Writing – review and editing; formal analysis. **Laura Creusot:** Writing – review and editing; formal analysis. **Camille Dupuy:** Formal analysis; writing – review and editing. **Patrick Emond:** Writing – review and editing; formal analysis. **Pranvera Krasniqi:** Formal analysis; writing – review and editing. **Antonin Lamaziere:** Writing – review and editing; formal analysis. **Cyriane Oeuvray:** Writing – review and editing; formal analysis. **Dominique Rainteau:** Writing – review and editing; formal analysis. **Magali Svrcek:** Writing – review and editing; formal analysis. **Nathalie Rolhion:** Writing – review and editing; formal analysis. **Harry Sokol:** Writing – review and editing; formal analysis; methodology; supervision. **Sophie Georgin‐Lavialle:** Investigation; conceptualization; methodology; writing – review and editing; supervision.

## FUNDING INFORMATION

This research was supported by grants from La Société Nationale Française de Médecine Interne (SNFMI‐REMI) and La Filière de Santé des Maladies Auto‐Immunes et Auto‐Inflammatoires Rares (FAI2R). The funding bodies played no role in the design of the study, data collection, analysis, or interpretation of the data, nor in the writing of the manuscript.

## CONFLICT OF INTEREST STATEMENT

All authors declare no financial or non‐financial competing interests.

## Supporting information


Table S1.

Figure S1.

Appendix S1.

## Data Availability

The datasets generated and/or analyzed during the current study are not publicly available due to privacy concerns, as the study involves a very rare disease with a limited number of patients, which poses a risk of patient re‐identification. However, the data are available from the corresponding author upon reasonable request.
