# Supplementary figures and images for: Functional changes in the gut microbiota are associated with the intestinal phenotype in A20 haploinsufficiency
Source: Pediatr Allergy Immunol. 2026 Apr 16;37(4):e70343. doi: 10.1111/pai.70343 (PMC13086603; doi:10.1111/pai.70343)

## Slide 1
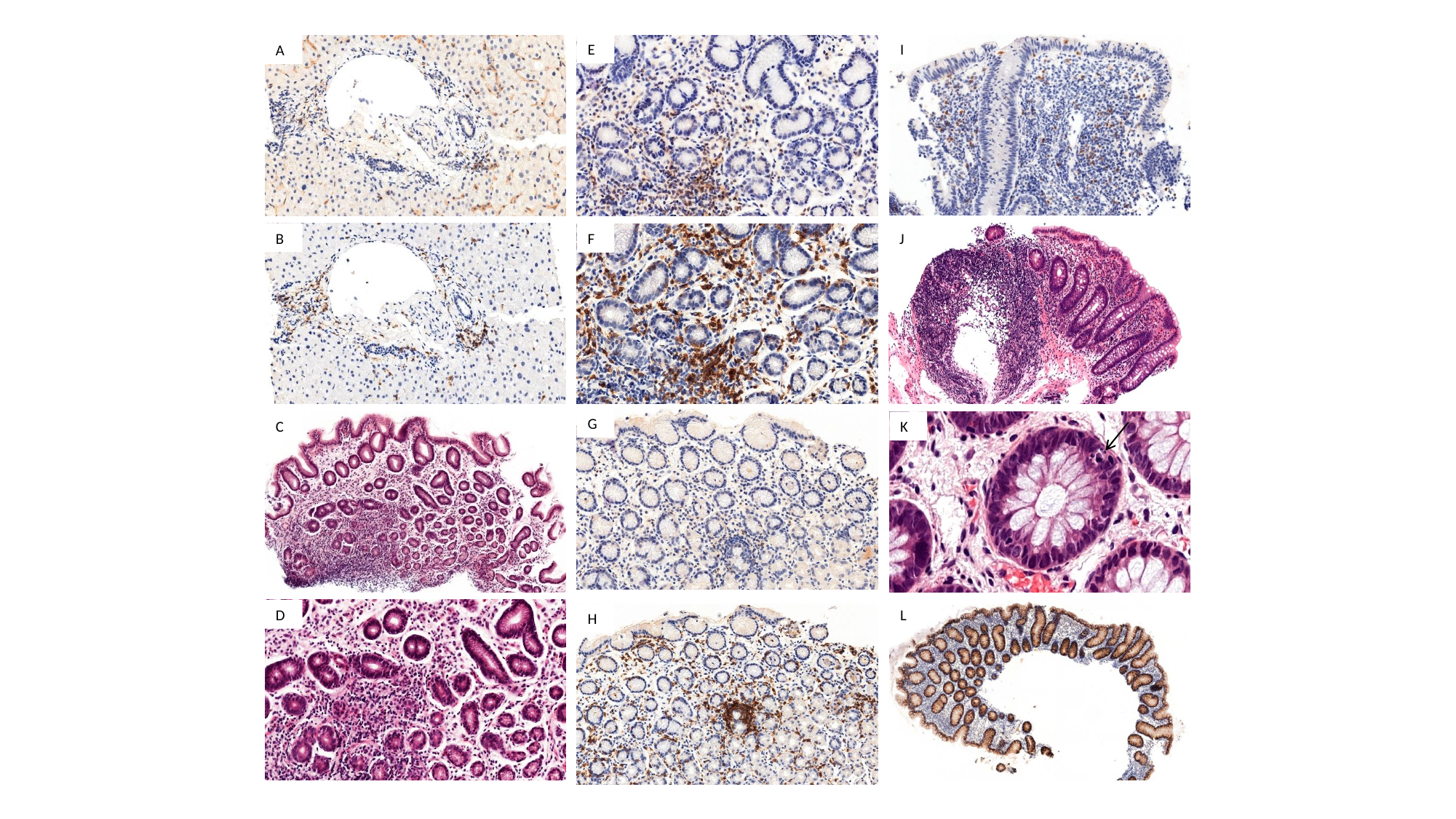

E
I
J
K
L
A
B
C
D
F
G
H

Supplement: Supplementary file 2 — Figure S1. [file PAI-37-e70343-s001.pptx]
