# Supplementary material for: Functional changes in the gut microbiota are associated with the intestinal phenotype in A20 haploinsufficiency
Source: Pediatr Allergy Immunol. 2026 Apr 16;37(4):e70343. doi: 10.1111/pai.70343 (PMC13086603; doi:10.1111/pai.70343)
Supplement: Supplementary file 3 — Appendix S1. [file PAI-37-e70343-s003.docx]

**Supplementary methods**

**Histological analysis**

Histopathological evaluation with HE staining recorded all relevant changes at each biopsy site, including the type and intensity of inflammatory infiltrate, signs of chronic injury, and specific features such as apoptosis (see supplementary data with detailed histopathological examination for each biopsy site). CD4 and CD8 staining were performed on all biopsy samples (stomach, ileum, colon, and liver) with an estimation of positive cell counts; CD4/CD8 ratio was evaluated as lower or higher than 1. MUC2 protein expression was also assessed in colonic biopsies.

The biopsy samples were fixed in formalin and paraffin embedded. Three-micrometer sections were prepared and stained with hematoxylin-eosin (HE). An immunohistochemical analysis was conducted using an automated immunohistochemistry system with antibodies targeting CD4 (clone 4B12, LEICA), CD8 (clone 4B11, LEICA), MUC2 (clone CCP58, AGILENT) at Saint-Antoine Hospital, using Leica platform, as well as NF-KB p-65 antibody (D14E12, Cell Signalling) at Caen-Normandie Hospital. Histopathological evaluation with HE staining recorded all relevant changes at each biopsy site, including the type and intensity of inflammatory infiltrate, signs of chronic injury, and specific features such as apoptosis (see supplementary data with detailed histopathological examination for each biopsy site). CD4 and CD8 staining were performed on all biopsy samples (stomach, ileum, colon, and liver), with an estimation of positive cell counts; CD4/CD8 ratio was evaluated as lower or higher than 1. MUC2 protein expression was also assessed in colonic biopsies.

For each biopsy site, various histopathological features were assessed, as detailed below. In gastric, ileal, and colon biopsies, the type and severity of inflammation were evaluated, including chronic lympho-plasmacytic inflammation, active inflammation (presence of neutrophils), high eosinophil count, and presence of lymphoid nodules. The degree of inflammation was categorized as mild, moderate, or severe, and the presence of erosion or ulceration was noted.

Specifically for gastric biopsies, glandular atrophy and the presence or absence of intestinal metaplasia were also assessed. In ileal and colonic biopsies, alterations in mucosal architecture, such as villous atrophy and crypt distortion, were recorded.

In liver biopsy examinations, histological features such as the type and severity of portal inflammation, lobular inflammation, apoptotic bodies, hepatocyte ballooning, steatosis and fibrosis were evaluated

**DNA extraction and sequencing**

Stool samples were resuspended in MGIEasy Stool Sample Collection Kit (1000005265 / 1000003702) and DNA extraction was performed using MagPure Stool DNA LQ Kit (384 RXN). 700µl stool sample suspension were transferred to 2.0ml deepwell plates containing MagPure grinding beads using MGI-STP7000, and extraction was performed using MGI-SP960 automation robot. Cell lysis was performed by beat-beating plate for 1min at 1600 rpm and thermal lysis at 650C for 20min. For DNA purification, 340µl of sample were used.

DNA libraries were prepared using MGIEasy FS DNA Library Prep Set (1000006988), circularized using MGIeasy circularization module V2.0 (1000005260) and sequenced on DNBSEQ-T7 using High-throughput Sequencing Set (FCL PE150) (1000016106).

Raw sequence data are accessible in the European Nucleotide Archive (accession number PRJNA951620).

**Read processing and quality control**

Reads quality was checked with FastQC (version 0.11.9), and low-quality reads and sequencing adapters were removed using Trimmomatic [1] (version 0.39). Reads shorter than 75 base pairs were discarded. Host-reads were removed using KneadData with default parameters (version 0.10.0; http://huttenhower.sph.harvard.edu/kneaddata) by mapping reads to the Homo sapiens reference database [2] (build hg37dec_v0.1).

**Microbiota analysis**

The samples were taxonomically profiled using MetaPhlAn 4 [3]. For the subsequent analyses (except for alpha-diversity calculations), the abundance of each taxon present in a sample was normalized using the relative method to allow sample-to-sample comparison. Taxa whose average abundance and prevalence were less than 0.1% and 3%, respectively, were discarded.

Functional potential analysis of the metagenomic samples (pathway profiles and gene-family abundances) was performed using HUMAnN3 [4] (version v3.0.0, UniRef database release 07-2021).

Principal coordinates analyses (PCoA) were carried out on the Bray-Curtis dissimilarity matrices constructed from the abundance of species and pathways. Communities that emerged were verified using a PERMANOVA test with Vegan package [5] (version 2.5-7). Figures were plotted with the ggplot2 [6] and ComplexHeatmap [7] packages.

Multivariable association between microbial community abundance and timepoint was examined with MaAsLin2 [8]. Age and sex factors were included in each analysis.

Spearman correlation analyses were conducted to associate metabolites and microbiota species using the R package energy (version 1.7-8). Correlations with adjusted p-values < 0.1 (Benjamini-Hochberg Procedure) were considered significant.

Estimated taxonomy of unannotated bacteria are described in supplementary table 1.

**Targeted metabolomics**

Fecal bile acids were analyzed in stool samples using a LC-20ADXR (Shimadzu, Kyoto, Japan) chromatographic system in tandem with a linear ion trap quadrupole MS/MS spectrometer QTRAP 5500 system (SCIEX, Ontario, Canada). Samples were assayed as previously described [9].

Fecal SCFAs were quantified on an Agilent 8890 GC system in tandem with an Agilent 5977B MS (Agilent technologies, Santa Clara, CA, USA). Extraction and quantification were previously described [10].

Fecal tryptophan metabolites were quantified as previously described [11].

**Statistical analysis for fecal metabolite levels comparison.**

The categorical variables were analyzed by using the chi-square test or Fisher’s exact probability test as appropriate. Continuous variables in two or three groups were compared using the Mann-Whitney test or the Kruskal-Wallis test, respectively. The significance level was set as p<0.05 for all analyzed data. Statistical analysis was performed with the online application EasyMedStat (version 3.21.5 ; www.easymedstat.com).

1. Bolger AM, Lohse M, Usadel B. Trimmomatic: a flexible trimmer for Illumina sequence data. Bioinformatics. 2014 Aug 1;30(15):2114–20.

2. Rosenbloom KR, Armstrong J, Barber GP, Casper J, Clawson H, Diekhans M, et al. The UCSC Genome Browser database: 2015 update. Nucleic Acids Res. 2015 Jan 28;43(D1):D670–81.

3. Blanco-Míguez A, Beghini F, Cumbo F, McIver LJ, Thompson KN, Zolfo M, et al. Extending and improving metagenomic taxonomic profiling with uncharacterized species using MetaPhlAn 4. Nat Biotechnol. 2023 Nov;41(11):1633–44.

4. Beghini F, McIver LJ, Blanco-Míguez A, Dubois L, Asnicar F, Maharjan S, et al. Integrating taxonomic, functional, and strain-level profiling of diverse microbial communities with bioBakery 3. Turnbaugh P, Franco E, Brown CT, editors. eLife. 2021 May 4;10:e65088.

5. Dixon P. VEGAN, a package of R functions for community ecology. J Veg Sci. 2003;14(6):927–30.

6. Wickham H. ggplot2 [Internet]. Cham: Springer International Publishing; 2016 [cited 2024 Aug 20]. (Use R!). Available from: http://link.springer.com/10.1007/978-3-319-24277-4

7. Gu Z. Complex heatmap visualization. iMeta. 2022;1(3):e43.

8. Mallick H, Rahnavard A, McIver LJ, Ma S, Zhang Y, Nguyen LH, et al. Multivariable association discovery in population-scale meta-omics studies. PLoS Comput Biol. 2021 Nov;17(11):e1009442.

9. Humbert L, Maubert MA, Wolf C, Duboc H, Mahé M, Farabos D, et al. Bile acid profiling in human biological samples: comparison of extraction procedures and application to normal and cholestatic patients. J Chromatogr B Analyt Technol Biomed Life Sci. 2012 Jun 15;899:135–45.

10. Mahdi T, Desmons A, Krasniqi P, Lacorte JM, Kapel N, Lamazière A, et al. Effect of Stool Sampling on a Routine Clinical Method for the Quantification of Six Short Chain Fatty Acids in Stool Using Gas Chromatography-Mass Spectrometry. Microorganisms. 2024 Apr 19;12(4):828.

11. Lefèvre A, Mavel S, Nadal-Desbarats L, Galineau L, Attucci S, Dufour D, et al. Validation of a global quantitative analysis methodology of tryptophan metabolites in mice using LC-MS. Talanta. 2019 Apr 1;195:593–8.
